# Hope as a protective factor for cognitive difficulties during the COVID-19 pandemic

**DOI:** 10.15761/fwh.1000186

**Published:** 2020-06-02

**Authors:** Emily Hicks, Craig McFarland

**Affiliations:** Department of Psychology at the University of Montana, Missoula, USA

**Keywords:** hope, cognition, COVID-19, women

## Abstract

The COVID-19 pandemic has had many negative outcomes, including problems of cognition; however, the degree to which individuals have noticed cognitive difficulties has varied. Protective factors that buffer against cognitive difficulties in women should be explored as women have faced great changes in the pandemic, including unemployment, increases in unpaid care work, increases in gender-based violence, and health concerns. For this reason, the present study sought to determine if hope acts as a protective factor for perceived problems of cognition. Using an online survey measuring aspects of cognitive functioning and hopefulness, results indicate that women with low hope report greater negative impacts of the COVID-19 pandemic on school and work, greater difficulties working from home, and more problems with attention, memory, and concentration than women with higher levels of hope. The findings suggest that hope may represent a protective factor that lessens the impact of the COVID-19 pandemic on perceived cognition.

## Introduction

The COVID-19 pandemic is a worldwide health crisis that has affected people’s work, schooling, finances, relationships, mental health, and virtually all aspects of life [1]. People are grieving the loss of loved ones, loss of important events, and loss of companionship. At the same time, communities are banding together to find innovative ways to stay connected, protect at-risk individuals, and support local businesses. It will take years to understand the full impacts of COVID-19, but it is important to understand the immediate responses to and outcomes of this crisis to learn how to meet the needs of our communities now and in future times of uncertainty. Factors that may buffer against harmful cognitive and behavioural impacts of COVID-19 should be explored to understand the variations in outcomes of COVID-19, as well as the protective role of these factors in times of crises. These factors are especially important to consider in women, as the COVID-19 pandemic has had a disproportionate effect. For example, a recent Centre for American Progress report noted that four times as many women as men were forced to leave their jobs in September, likely due to childcare and school crises [2], though census data shows that women with and without children have faced higher rates of unemployment than men during the pandemic [3]. On top of the negative economic impacts of job loss and increased amount of unpaid care work, women have also faced health concerns and increases in gender-based violence during stay-at-home orders [4]. These substantial life changes have impacted women to varying degrees. Protective factors that may lessen the intrusiveness and harmfulness of COVID-19 on women’s lives should be explored to better meet women’s needs during this time of crisis.

### Impacts on cognition

It is clear that, the COVID-19 pandemic has caused disruption in both work and school environments. In response to the virus, there were sweeping institutional closures and transitions to remote learning and working environments. Many individuals have been asked to not only work from home but also to contribute more directly to their children’s education, creating a demanding daily cognitive load. University students were asked to rapidly transition to a remote learning environment, though many prefer in-person instruction. All these demanding tasks may impact cognitive functioning.

One study found a 7% prevalence rate of Posttraumatic Stress Syndrome (PTSS) in the areas of China that were most heavily impacted by the new coronavirus, with negative alterations in cognition being a frequent symptom [1]. These early findings suggest that impaired cognition may be a negative outcome that will be observed worldwide. Although research regarding COVID-19 is limited, an extensive body of research has established the negative impacts of high stress on cognition [5–8]. This research coupled with early findings revealing the negative cognitive outcomes associated with COVID-19 highlight the importance of studying the relations between stress and COVID-19, and their impact on cognition.

### Emotional and affective outcomes

COVID-19 brought about an uncertain time for women worldwide, with finances, relationships, and access to resources changing swiftly. These widespread changes have led to negative emotional outcomes. Studies have found that both the public and medical workers are experiencing vicarious traumatization related to the COVID-19 pandemic [9]. This problem is exacerbated by the fact that many individuals may not know how to access mental health resources during a time where most states have implemented stay-at-home orders. Additionally, the current pandemic has impacted both adults [10] and children [11]. Research has found an increase in anxiety symptoms, increase in depressive symptoms, and poorer sleep quality in people impacted by COVID-19 [12]. These findings are disturbing as each of those factors can contribute to an overall sense of decreased well-being and impaired cognitive functioning. These negative emotional effects are impacting communities across the globe. In Italy, two infected Italian nurses completed suicide, and it is speculated that they feared spreading the virus to patients [13]. Clearly the emotional and affective impacts of COVID-19 should be of utmost concern.

Although numerous negative emotional outcomes have been noted, there may also be positive effects. For example, individuals may feel a sense of gratitude for their loved ones, joy in having more time with family, or satisfaction in learning a new hobby or skill. People may find that they continue to feel hopeful despite negative impacts of the pandemic. Previous research has noted that hope is protective by encouraging low levels of negative emotions and aiding with recovery from stress [14]. Individuals high in hope demonstrate resilience and positive responses to stress [14]. Research has found that increased levels of hope predict lower levels of depression and anxiety [15]. Thus, while all women have been impacted by the COVID-19 pandemic, some may find ways to cope with the stress in the forms of gratitude and joy, new hobbies, and skills, or maintaining hopefulness.

### Present study

Given the limited research regarding COVID-19 responses and outcomes, lack of studies regarding potential protective factors of problematic COVID-19 impacts, and data noting the benefits of hopefulness during times of high stress, the current study seeks to investigate differences in cognitive outcomes with regard to varying degrees of hopefulness in U.S. women. The present study used a survey to quantitatively examine COVID-19 responses and outcomes.

## Materials and method

### Participants

Participants consisted of adults aged 18-years-old and older who identify as female. Participants self-selected to complete the online survey, which was advertised on social media platforms, including Facebook, Twitter, and Instagram. The survey took approximately ten minutes and no incentives were given to complete the survey.

### Assessments and measures

#### Cognitive outcomes

The implications of COVID-19 on cognition were assessed using six items designed for this study. The items can be seen in Table 1. Items were scored on a 5-point Likert scale ranging from 1 (strongly disagree) to 5 (strongly agree) and were found to have an internal consistency of 0.822.

#### Hopefulness

Hopefulness about the future was measured with a single item, which can also be viewed in Table 1. This item was scored on a 5-point Likert scale ranging from 1 (strongly disagree) to 5 (strongly agree).

#### Statistical analyses

In order to make comparisons of cognition across different levels of hope, hopefulness was converted to a binary construct where Group 1 consists of women who responded to the hopefulness item with strongly disagree, disagree, or neither agree nor disagree, and Group 2 consists of women who responded to the hopefulness item with agree or strongly agree. Thus, Group 1 can be considered women with low hopefulness, and Group 2 can be considered women with high hopefulness.

An independent samples t-test was used to compare self-reported cognition between women with low hope (*N* = 54) and women with high hope (*N* = 74). It should be noted that three participants did not respond to the item COG_2, two participants did not respond to the item COG_4, and one participant did not respond to the items COG_5 and COG_6. Therefore, the number of participants in each group changed slightly in these items.

## Results

The sample consisted of individuals who identify as female. The majority (36.0%) of participants were between the ages of 18 to 25, and the majority (89.7%) of participants had either a bachelor’s degree or graduate degree. The sample was 87.5% White and 16.2% of participants identified as immunocompromised. Further information regarding participant demographics is presented in table 2.

### COG_1 and hope

**Figure 1.**
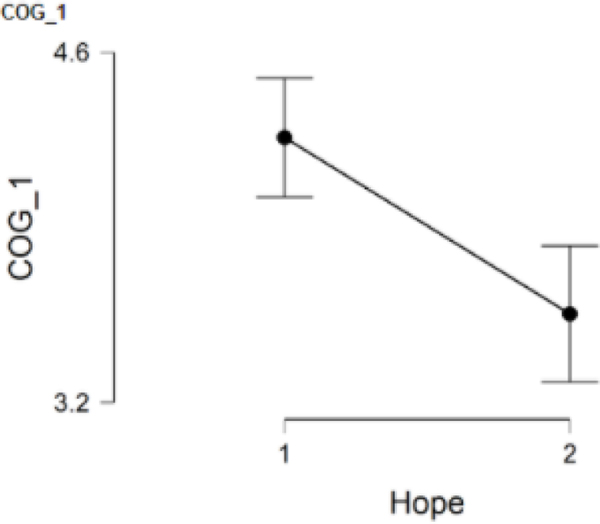
COG_1 and hope

COG_1, which asked participants to rate the degree to which their schooling or work had been negatively affected by the pandemic, was answered by 128 participants with 54 participants falling in the low hope group (Group 1) and 74 participants falling in the high hope group (Group 2). When comparing the low hope group (*M* = 4.3, *SD* = 0.9) to the high hope group (*M* = 3.6, *SD* = 1.2), the low hope group had statistically significant higher scores on COG_1, *t*(126) = 3.73, *p* = 0<.001. Furthermore, Cohen’s effect size value (*d* = 0.67) suggested a moderate to high practical significance. This finding indicates that women in Group 1 reported greater negative effects in school and work due to the pandemic (Figure 1).

### COG_2 and hope

**Figure 2.**
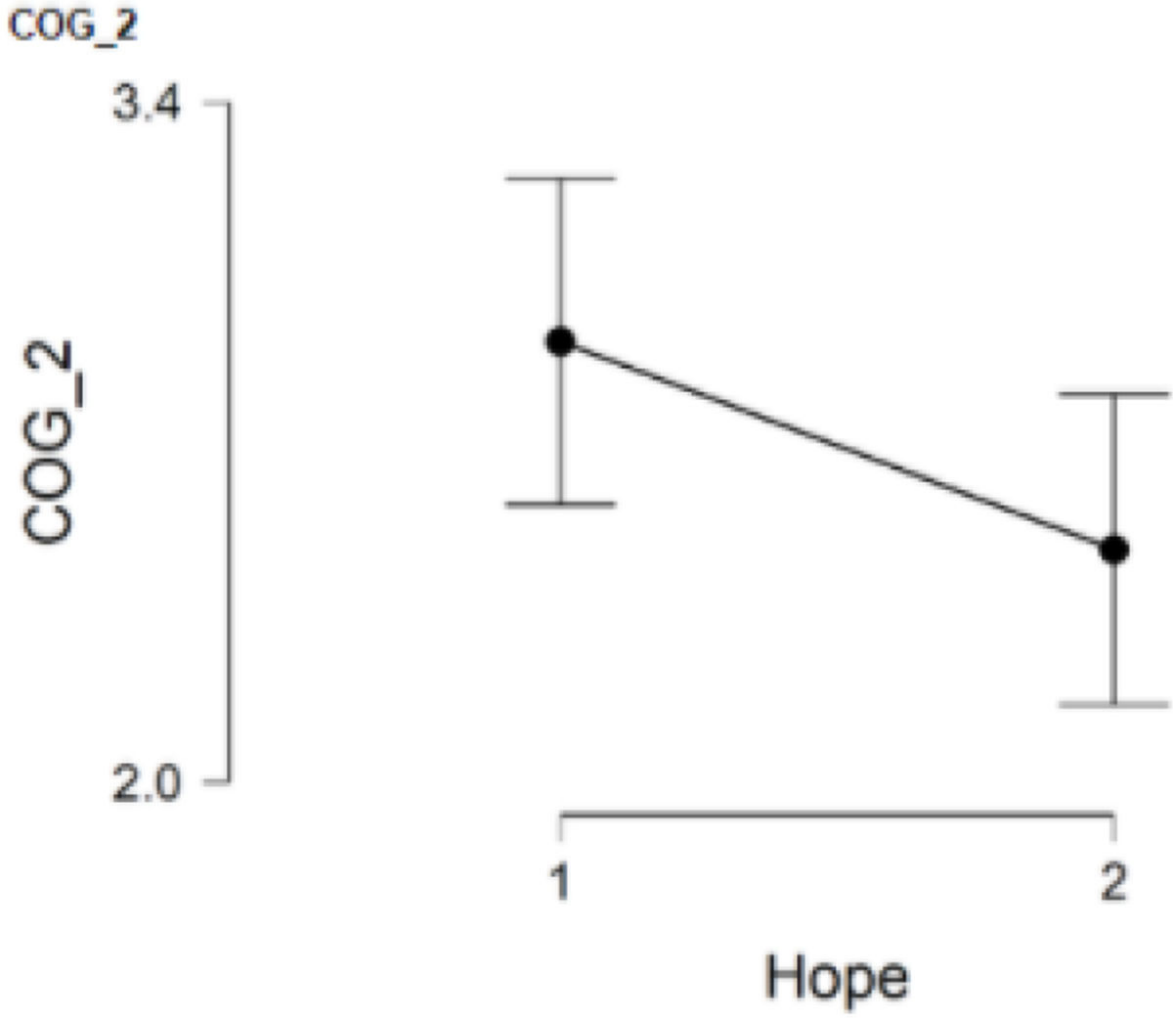
COG_2 and hope.

COG_2, which asked participants to rate whether or not they had to drop school or work activities due to COVID-19 related stressors, was answered by 125 participants with 54 participants in the low hope group (Group 1) and 71 participants in the high hope group (Group 2). Results suggested no statistically significant differences between Group 1 (*M* = 2.9, *SD* = 1.2) and Group 2 (*M* = 2.5, *SD* = 1.4), *t*(123) = 1.82, *p* = .071. Additionally, Cohen’s effect size (*d* = 0.33) suggested small practical significance; however, the low hope group tended to report a greater number of dropped school and work activities during the pandemic (Figure 2).

### COG_3 and hope

**Figure 3.**
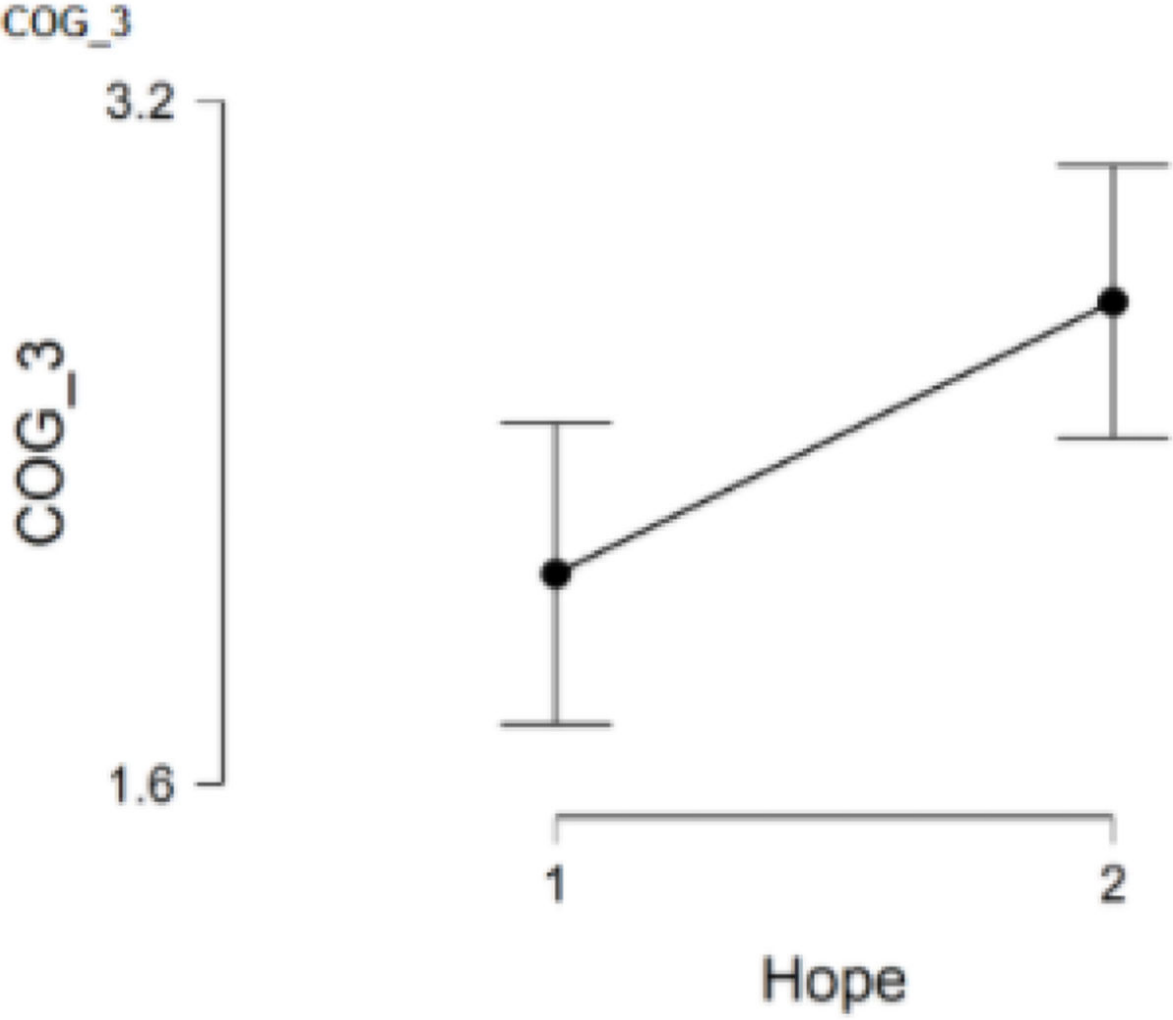
COG_3 and hope.

COG_3 asked participants to rate the degree to which they felt they worked as well from home as in person and was answered by 128 participants with 54 participants in Group 1 and 74 participants in Group 2. The low hope group (*M* = 2.1, *SD* = 1.3) had statistically significantly lower scores than the high hope group (*M* = 2.7, *SD* = 1.4), *t*(126) = −2.65, *p* = .009. Further, Cohen’s effect size value (*d* = −0.48) suggested moderate practical significance. This finding indicates that those in the low hope group tended to feel more strongly that they did not work as well from home as in person when compared to the high hope group (Figure 3).

### COG_4 and hope

**Figure 4.**
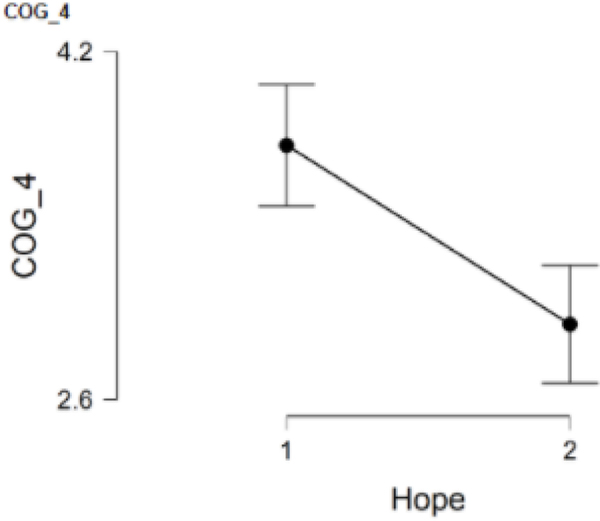
COG_4 and hope.

COG_4 had participants rate the degree to which their attentional abilities were negatively impacted by COVID-19, and 126 participants responded to this item. Group 1 had 52 participants and Group 2 had 74 participants. When comparing the low hope group (*M* = 3.8, *SD* = 1.0) to the high hope group (*M* = 2.9, *SD* = 1.2), results revealed statistically significantly higher scores in the low hope group, *t*(124) = 4.12, *p* = <.001. Cohen’s effect size (*d* = 0.75) suggested a large practical significance as well. This result suggests that women in the low hope group perceive more problems of attention during the pandemic than women in the high hope group (Figure 4).

### COG_5 and hope

**Figure 5.**
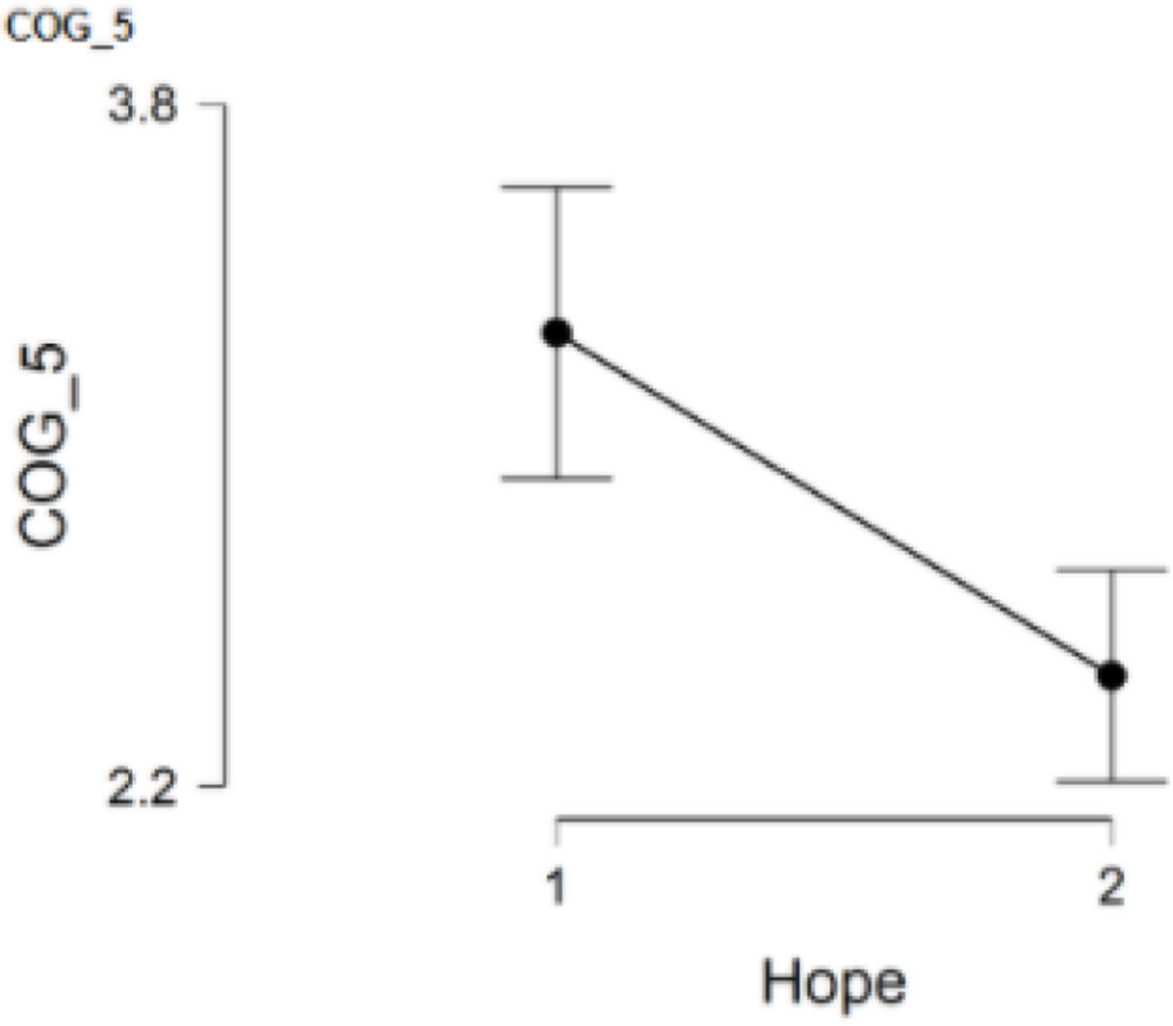
COG_5 and hope.

This item asked participants to rate the degree to which they noticed increased memory difficulties since the pandemic began. COG_5 was answered by 127 participants with 53 participants in the low hope group and 74 participants in the high hope group. Results suggested a statistically significant difference between Group 1 (*M* = 3.3, *SD* = 1.2) and Group 2 (*M* = 2.5, *SD* = 1.0), with the low hope group having higher scores on COG_5, *t*(125) = 3.90, *p* = <.001. Furthermore, Cohen’s effect size value (*d* = 0.70) suggested a moderate to high practical significance. Responses to this item indicate that women in the low hope group have noticed greater memory difficulties than women in the high hope group since the beginning of the pandemic (Figure 5).

### COG_6 and hope

**Figure 6.**
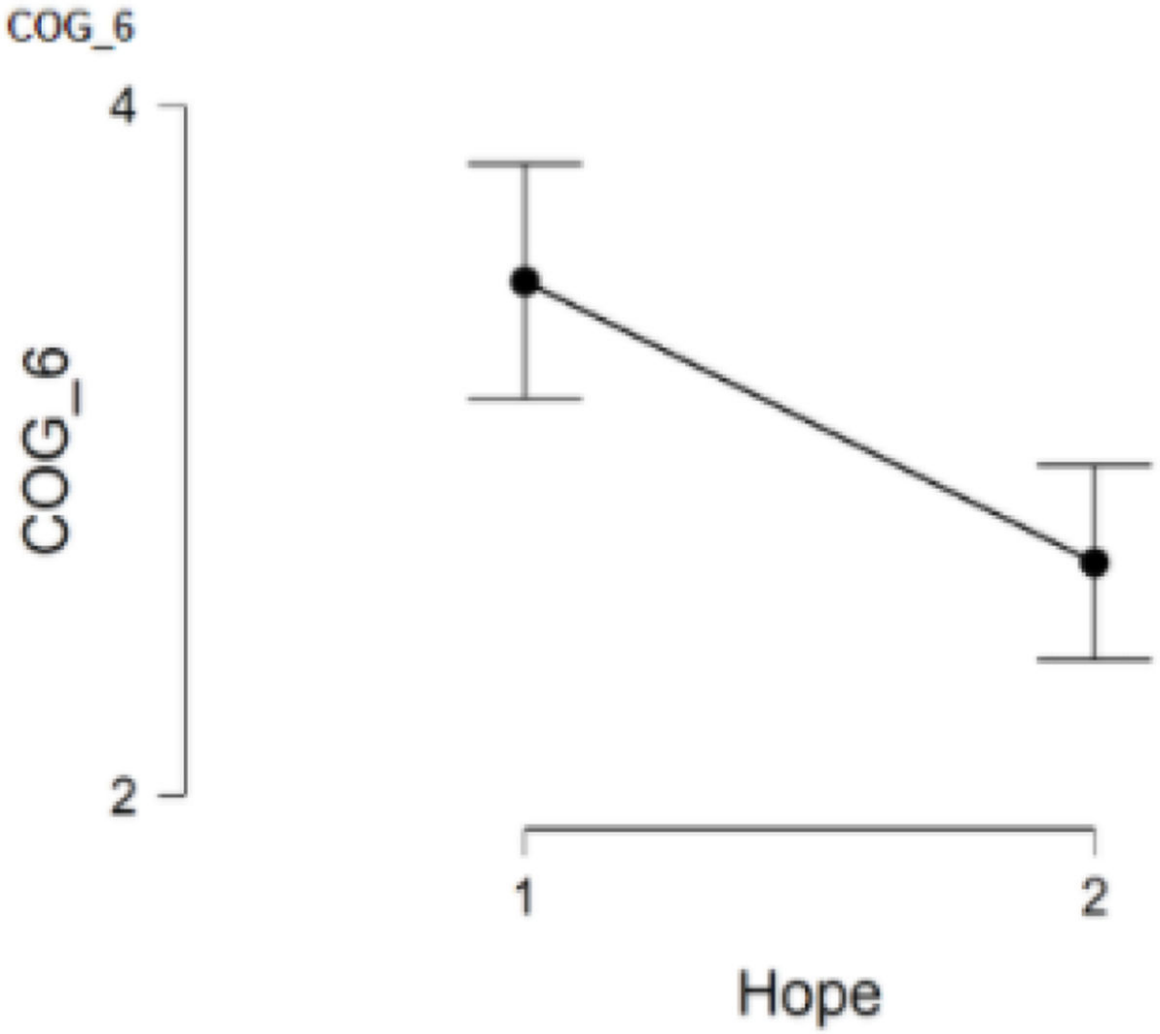
COG_6 and hope.

The final cognitive outcome item had participants rate the degree to which they observed negative impacts on task concentration since the beginning of the pandemic. This item, COG_6, was answered by 127 participants with 53 participants in Group 1 and 74 participants in Group 2. When comparing the low hope group (*M* = 3.5, *SD* = 1.2) to the high hope group (*M* = 2.7, *SD* = 1.2), the results indicate statistically significant differences between the groups with the low hope group having higher scores on COG_6, *t*(125) = 3.70, *p* = <.001. Cohen’s effect size (*d* = 0.67) suggested a moderate to high practical significance. This finding suggests that women in the low hope group have noticed greater problems with concentration during COVID-19 than women in the high hope group. Results from all items can be seen in table 3 (Figure 6).

## Discussion

The purpose of this study was to determine if hopefulness buffers against cognitive difficulties during the COVID-19 pandemic. Results from the present study suggest that hope acts as a protective factor for perceived problems of cognition. Women who reported low levels of hope (Group 1) reported greater negative impacts in school and work, greater difficulties working from home, and more problems with attention, memory, and concentration during the COVID-19 pandemic than women who reported high levels of hope (Group 2). These findings were both statistically and practically significant, suggesting that difficulties on cognition were noticeable and impactful in women’s daily lives. Level of hope was not related to the need to drop school or work activities.

These results are consistent with expectations as women who are more discouraged about the future may be more distracted during school and work due to worry, be more attentive to negative impacts, and may consider life transitions as difficult and harmful. On the contrary, women with greater hope about the future may perceive transitions as a challenge, but not harmful, and may be better able to focus on tasks as they are not preoccupied with future concerns. The findings support that women with higher hope have a protective factor that lessens the impact of the COVID-19 pandemic on perceived cognition.

### Limitations

This study is not without limitations, and results of the study should be interpreted with consideration to the unique characteristics of the sample. The similarity of the sample demographics limits the ability to generalize to the wider population. The majority of the participants were White and had received higher education. Women from minority groups should be given great consideration in future research, as early research suggests that these women may face the greatest economic impacts of COVID-19 [2], which may affect levels of hope. Additionally, the present study did not consider the presence of potential mood disorders, which may impact both hopefulness and cognition. Future research should examine the interactions between these factors. Data from this study was self-reported, and future research should use established measures of cognition to determine if perceived cognitive difficulties result in lower scores on cognitive assessments. Finally, the number of life alterations due to COVID-19, such as changes in employment and childcare, were not considered in the present study. Future work should determine if the number of life transitions affects women’s levels of hope.

## Conclusion

The established benefits of hopefulness on cognition are important as they provide insight into why the pandemic has produced varying impacts on individuals perceived cognitive functioning. Additionally, this knowledge may encourage women to nurture hope even in times of crisis. There are evidence-based mechanisms used to increase hope, such as mindfulness and re-evaluating barriers as challenges, which may not only encourage positive affect but also positively impact cognitive abilities. In a time where it is easy to focus on negative outcomes, attention should be given to the resilience that women have displayed in the face of adversity. The results of this study highlight this resilience and the protective benefits of hope on cognitive abilities in uncertain times.

## Figures and Tables

**Table 1.** Cognitive and hopefulness items

Item Name	Item Content
Cognitive Items	
COG_1	My school / work has been negatively affected by the pandemic.
COG_2	I had to drop a school course or work-related activities due to COVID-19 related stressors
COG_3	I learn / work from home as well as I do in person.
COG_4	My attentional abilities have been negatively affected by the pandemic.
COG_5	I am having a harder time remembering things now than before the COVID-19 crisis started.
COG_6	I am not able to concentrate on tasks as well since quarantine began.
Hopefulness Item	
Hope	I am hopeful about the future following COVID-19.

**Table 2.** Participant demographics.

Variable	*N*	Percentage
**Age**		
18–25	49	36.0%
26–35	28	20.6%
36–55	39	28.7%
56–69	16	11.8%
70+	4	2.9%
**Education**		
Less than high school	0	0.0%
High school graduate	3	2.2%
Some college	10	7.4%
2-year degree	1	0.7%
4-year degree	63	46.3%
Graduate degree	59	43.4%
**Race/Ethnicity**		
American Indian/Alaska Native	7	5.1%
Asian	1	0.7%
Black/African American	1	0.7%
Hispanic/Latinx	7	5.1%
Native Hawaiian/Other Pacific Islander	1	0.7%
White / Caucasian	119	87.5%
**Immunocompromised status**		
Yes	22	16.2%
No	113	83.1%

Note. N = 136.

**Table 3.** Independent samples t-test. Student’s t-test.

	*t*	*df*	*p*	Cohen’s *d*
COG_1	3.729	126	<.001*	0.667
COG_2	1.824	123	0.071	0.329
COG_3	−2.651	126	0.009*	−0.475
COG_4	4.123	124	<.001*	0.746
COG_5	3.896	125	<.001*	0.701
COG_6	3.698	125	<.001*	0.665

* = p < 0.01.
